# A Real-Time Recording Model of Key Indicators for Energy Consumption and Carbon Emissions of Sustainable Buildings

**DOI:** 10.3390/s140508465

**Published:** 2014-05-14

**Authors:** Weiwei Wu, Huanjia Yang, David Chew, Yanhong Hou, Qiming Li

**Affiliations:** 1 School of Civil Engineering, Southeast University, No.2 Si Pai Building, Nanjing 210096, China; E-Mail: njlqming@163.com; 2 Department of Computer Science, Loughborough University, N.3.28, Haslegrave Building, Leicestershire LE11 3TU, UK; E-Mail: h.yang@lboro.ac.uk; 3 School of Civil and Environmental Engineering, Nanyang Technological University, 639798, Singapore; E-Mail: caschew@ntu.edu.sg; 4 School of International Pharmaceutical Business, China Pharmaceutical University, Nanjing 211198, China; E-Mail: houyanhong12@126.com

**Keywords:** real time, key indicators, RFID-based, sustainable building

## Abstract

Buildings' sustainability is one of the crucial parts for achieving urban sustainability. Applied to buildings, life-cycle assessment encompasses the analysis and assessment of the environmental effects of building materials, components and assemblies throughout the entire life of the building construction, use and demolition. Estimate of carbon emissions is essential and crucial for an accurate and reasonable life-cycle assessment. Addressing the need for more research into integrating analysis of real-time and automatic recording of key indicators for a more accurate calculation and comparison, this paper aims to design a real-time recording model of these crucial indicators concerning the calculation and estimation of energy use and carbon emissions of buildings based on a Radio Frequency Identification (RFID)-based system. The architecture of the RFID-based carbon emission recording/tracking system, which contains four functional layers including data record layer, data collection/update layer, data aggregation layer and data sharing/backup layer, is presented. Each of these layers is formed by RFID or network devices and sub-systems that operate at a specific level. In the end, a proof-of-concept system is developed to illustrate the implementation of the proposed architecture and demonstrate the feasibility of the design. This study would provide the technical solution for real-time recording system of building carbon emissions and thus is of great significance and importance to improve urban sustainability.

## 1. Introduction

The global climate is being affected by the emissions of greenhouse gases, of which the most significant is carbon dioxide (CO_2_) from human development [1–3]. Buildings are one of the main factors of energy consumption and greenhouse gas emissions and thus it is of great urgency in regards to environment protection and sustainable development to reduce energy consumption and carbon dioxide emissions from buildings [4,5].

Buildings' sustainability is one of the crucial parts of achieving urban sustainability. Applied to buildings, life-cycle assessment encompasses the analysis and assessment of the environmental effects of building materials, components and assemblies throughout the entire life of the building construction, use and demolition. Estimation of carbon emissions is essential and crucial for an accurate and reasonable life-cycle assessment.

It is pointed out that accurate analysis of urban carbon cycle and its interactions with other global or regional ecosystems is going to be crucial for predictions of future trajectories of atmospheric CO_2_ concentrations and global climate change [2]. However, some crucial indicators in the process of calculation such as the quantities of certain materials, transport distance, electricity use and so on are either over-estimated or under-estimated in the analysis as no official bill of materials prepared by the designers or builders of the buildings is typically available, causing both the uncertainty of the calculation results and the comparison results based on different building structures and even different stages [1,5–7].

Some efforts have been made on the calculation of energy consumption and carbon emissions of buildings. You *et al.* set up an integrated model to analyse the carbon emissions of an urban building system during its life cycle [2]. Wu *et al.* developed a life cycle assessment model for an office building to assess its energy consumption and carbon emissions [5]. Gustavsson *et al.* used bottom-up analytical techniques to analyse the life cycle primary energy use and carbon dioxide (CO_2_) emissions of an eight-storey wood-framed apartment building including acquisition and processing of materials, on-site construction, building operation, demolition and materials disposal [1]. They showed that the building operation incurs the largest share of life cycle energy use, which becomes increasingly dominant as the life span of the building increases.

Cole provided a detailed examination of the energy and greenhouse emissions associated with the on-site construction of a selection of alternative wood/steel and concrete structural assemblies [7]. Gerilla *et al.* found that the emissions from carbon pollutants in the operation stage were the highest compared to the other lifecycle stages [8]. Carbon emissions from the operation stage constituted about 79% of the total emissions. The carbon emission from the construction stage was only about 12% of the total lifecycle while maintenance and disposal only had around 9% of the total carbon emissions [8].

Ortiz *et al.* brought together research on life cycle assessment (LCA) applied within the building sector and the present review had tried to compile and reflect the key milestones accomplished in LCA over the seven years within the building sector, from 2000 to 2007 [9]. Bribian *et al.* presented the state-of-the-art research regarding the application of life cycle assessment (LCA) in the building sector, providing a list of existing tools, drivers and barriers, potential users and purposed of LCA studies in this sector and the embodied energy can represent more than 30% of the primary energy requirement during the life span of a single house with a garage for one car [10].

Adalberth presented a method on how to calculate the energy use during the life cycle of a building [11]. Seo and Hwang divided the life cycle of a residential building into four stages (manufacturing, construction, operation, and demolition) for estimating CO_2_ emissions [12]. Blengini estimated carbon emissions derived from three distinct phases: pre-use, use and end-of-life [13]. Erlandsson and Borg divided LCA for buildings into a linear life cycle and a sequential life cycle [14]. Malmqvist *et al.* adopted a systematic approach guiding the user through the life cycle process and clarifying key issues that usually cause difficulty [15]. Chen *et al.* presented a low-carbon building evaluation framework detailing carbon emission account procedures for the life cycle of buildings in terms of nine stages of building construction, fitment, outdoor facility construction, transportation, operation, waste treatment, property management, demolition, and disposal for buildings [16].

Gerilla *et al.* assessed the environmental impacts of wooden type and steel reinforced concrete type of construction, showing that steel reinforced concrete (SRC) construction has a higher environmental impact compared to the wooden type of housing construction [8]. Yan *et al.* determined carbon emissions derived from manufacture and transportation of building materials, energy consumption of construction equipment, energy consumption for processing resources, and disposal of construction waste [17]. Hacker *et al.* studied embodied carbon emission of building materials and operation carbon emission, in which carbon emission originated from other stages of the life cycle of buildings were omitted, with operation carbon emission just focusing on that derived from energy used to operate appliances [18].

Jiang and Tovey proposed a series of low-carbon sustainability strategies including effective energy management, technical measures for energy conservation, renewable energy technologies, awareness raising and behavior change, and offsetting methods for Chinese buildings, while carbon emission costs for the implementation of these strategies were not accounted for [19]. Knudstrup *et al.* discussed a survey of different types of approaches to the sustainable design of building and showed example of new Danish housing projects that can minimize the use of energy for heating and cooling in the shape of detached houses [20].

Sathre and Gustavsson analyzed the relations between building material competitiveness and economic instruments for mitigating climate change and the results indicate that higher energy and carbon taxation rates increase the economic competitiveness of wood construction materials [21]. Nassen *et al.* used top-down input-output analysis to assess the magnitude of primary energy use and CO_2_ emissions linked to the production of buildings [22].

However, some key indicators are commonly used and mostly are estimated afterwards in the calculation process including quantity use of building material (diesel fuel, electricity, concrete, cement, brick, steel, timber, glass, plastic), transportation distance of building material (concrete, cement, brick, steel, timber, glass, plastic), energy consumption during construction-installation on-site process, electricity use for cooling, ventilating, lighting, and water supply during operation and so on [2,5]. Most of these indicators are estimated in the calculation process, as shown in Table 1. The accurate records of these key indicators based on real-time information are essential for a more accurate calculation and comparison of energy use and carbon emissions of buildings.

In a word, the gaps of extant research majorly lie in two aspects. One is the accurate recording of these key indicators based on real-time information. The other is the real-time transfer and sharing of these key indicators.

Addressing the need for more research into the integration and automatic recording of key indicators for a more accurate calculation and comparison, this paper aims to design a real-time recording method as well as transfer and sharing model of these crucial indicators from the perspective of whole life cycle concerning the calculation of energy consumption and carbon emissions of sustainable buildings based on a Radio Frequency Identification (RFID) system.

The organization of the paper is as follows: (1) Section 1 (Introduction) introduces the background and analyses the gaps of extant research; (2) Section 2 (Methodology) introduces the RFID and Wireless Sensor Networks (WSN) technology, which have the potential to fill extant gaps; (3) Section 3 (Data Requirement Analysis) investigates the data requirements of real-time recording, transfer and sharing of key indicators; (4) Section 3 to Section 6 cover the design of the concept model and the system architecture, as well as the illustration of implementation and demonstration system; (5) Section 7 (Discussion and Conclusions) is the summary of the whole paper.

This study provides a technical solution for a real-time recording model of key calculation and estimation indicators for energy consumption and carbon emissions and thus is of great significance and importance to improve building sustainability.

## 2. Methodology

RFID and WSN have the potential to be used to fill the gaps of accurately recording, real-time transfer and sharing of key indicators in the process of calculating the energy use and carbon emissions of buildings.

### 2.1. RFID Technology

The development of promising information technologies, such as RFID and WSN has prompted research into their application to construction industry. Automatic Identification (Auto-ID) technologies are a broad term describing technologies that enable machines to identify objects. The key for Auto-ID technologies is their automatic data capture ability. RFID is a type of Auto-ID technology that uses radio waves to automatically identify people or objects. Compared to other Auto-ID technologies the RFID provides a non-contact data transfer between the RFID tags and the reader/interrogators without the need for a strictly obstacle-free, line-of-sight reading. Some RFID tags allow the information carried to be rewritten, which improves the flexibility of the technology. Moreover, a RFID reader can read multiple tags simultaneously to improve efficiency [23].

The basic components of a typical RFID system include: the transponder or the tag, which is a microchip in which a unique serial code is stored and transmitted when necessary via an antenna attached; the RFID reader/interrogator, which is used to retrieve/receive the identify from the tags; there are generally three types of RFID tags, depending on the power source used, which are the active, passive and semi-passive/semi-active tags. Each has its own features and is suitable for certain types of applications. Among the various technologies, the passive RFID is the one that has become the focus of ID technology in the past decade. Because of its simple, powerless, low cost and reliable tag design, the passive RFID technology has the potential to be adopted in the large scale applications in which a massive number of tags need to be deployed.

RFID technologies represent efficient and low cost solutions for identifying and tracking of objects and personnel. They have been adopted in a wide range of applications, such as healthcare [24], manufacturing [25] and food industries [26]. RFID in construction applications is not a new idea. Lu *et al.* have summarized the research of RFID in construction [27]. Most of the research focuses on using RFID in the construction supply chain [28], construction quality inspection and management [29] and construction assets tracking [30–32].

### 2.2. WSN (Wireless Sensor Network) Technology

WSN is a type of *ad-hoc* wireless network composed of a large number of spatially distributed autonomous sensor nodes that interact with the physical world for remote monitoring and control applications [33–35]. The ZigBee technology, which is built based on the IEEE802.15.4 standard [36], is one of the most popular WSN standards [37]. Its specification is maintained by the ZigBee Alliance which consists of members from both academic and industry [38]. The ZigBee technology features a self-organized and multi-hop wireless network structure, which provides an easy and fast system implementation. The IEEE 802.15.4 standard defines the Physical and MAC layers, in which two device types, the Full Function Devices (FFDs) and Reduced Function Devices (RFDs) are used to form a low energy wireless network with a star topology. ZigBee defines a network layer upon the IEEE 802.15.4 structure. It introduces coordinator, router and end device as the three main types of network level devices that construct a wireless sensor network with mesh topology. The ZigBee coordinator is based on IEEE 802.15.4 FFD and is responsible for establishing the network, managing the joining of network devices and allocating network address blocks. A ZigBee network must have one and only one coordinator. The ZigBee router devices are also based on IEEE 802.15.4 FFD. They form the backbone of the ZigBee network and are responsible for the multi-hop data relay between network nodes. Both the ZigBee coordinator and the ZigBee routers can connect with multiple nodes in the ZigBee network so that a mesh network topology can be built. The ZigBee end devices are based on IEEE802.15.4 RFD. Each of them can connect to only one parent node, which must be either a coordinator or a router device. The end devices can be deemed as the leaf nodes of the network and can only be accessed through their parent nodes.

The ZigBee technology provides a reliable, low cost and easy to deploy wireless sensor network solution. In our research, we try to extend the application of ZigBee network systems. We consider that a ZigBee system not only transmits the sensory data from the end nodes deployed, but also has the potential of providing a wireless data transmission network structure from which the other types of information gathering devices, such as RFID readers, may also benefit. Actually, it has been recognized that there is a great potential in combining the ZigBee and RFID technologies to achieve a system that has the features from both sides. In our previous research, such an integrated system is called a ZigBee RFID Sensor Network. Various system architectures at the network level have been studied for general integrations of RFID and WSN [36,37].

## 3. Data Requirement Analysis

### 3.1. Life Cycle of a Building

**Figure 1.**
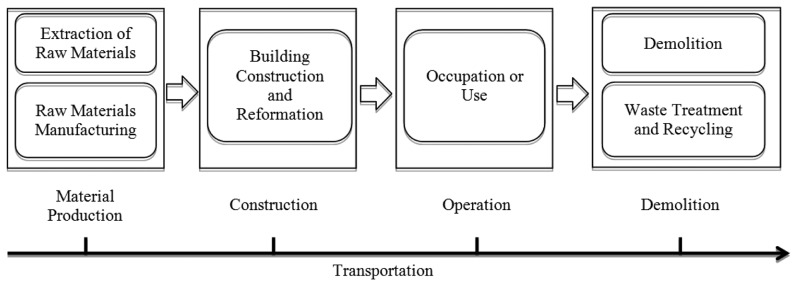
The life cycle phases of a building.

Gustavasson *et al.* pointed out that the life cycle of a building includes the extraction of raw materials, processing of raw materials into building materials, assembly of materials into a ready building, assembly of materials into a ready building, occupation or use, maintenance, demolition or disassembly of the building and disposal or re-use of the materials [1]. A life cycle of materials production, construction, operation and demolition is also used in some assessment [5]. Moreover, generally, there are five temporal phases including construction materials preparation, building construction and reformation, building operation, building demolition as well as wastes treatment and recycling [2]. In this research, a life cycle of four general stages of materials production, construction, operation and demolition are used when combining the stage of wastes treatment and recycling into demolition, as Figure 1 shows.

### 3.2. Analysis of Data Requirement for Real-Time Recording

**Figure 2.**
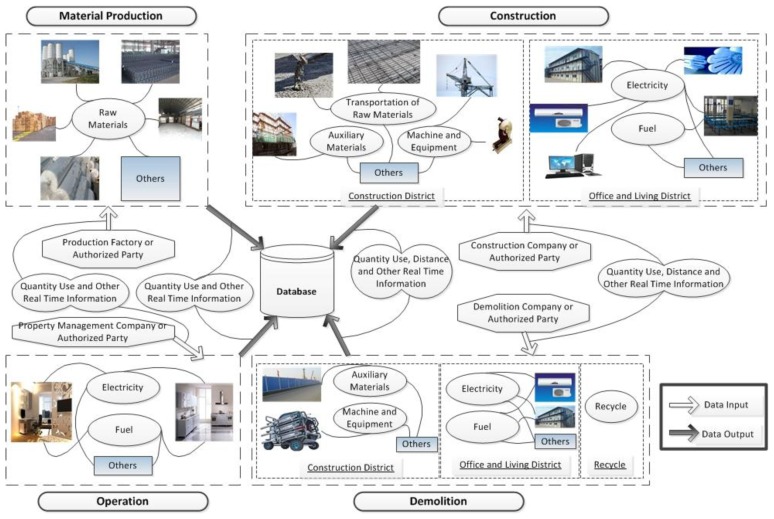
Model of data requirement for real-time recording.

A model of data requirement for real-time recording is illustrated in Figure 2.

In the stage of material production, the data of quantity of raw materials used, including concrete, cement, brick, steel, timber, glass, plastic and so on, is maintained by the production company, which is also required to exchange the data based on real-time information with a database that will be used for the backup and calculation of energy consumption and carbon emissions. Considering the nature of non-profitability of carbon emission at a company level, we expect such a database to be maintained by some authority department, construction association or authorized non-government organizations. The structure and details of the database is out of the scope of this paper.

When it comes to the stage of construction, the data of transportation distance of raw materials is maintained by the construction company and is also required to be exchanged with the database on a real-time basis. Moreover, the amount used and transportation distance of auxiliary materials that cannot be calculated together with the raw materials are also recorded. As far as the machines are concerned, including excavators, tower cranes, rollers and so on, the transportation distance and the amount of fuel and electricity used are recorded for future calculation. The transportation distance and the quantity use of fuel and electricity of equipment are also maintained based on real-time information such as cutting off machines, electric welding machines and lifts. For the office and living areas, the transportation distance of board room and others and the quantity use of fuel and electricity are also recorded based on real-time information. In the meantime, the quantity of raw materials and others that are not used on site will be recorded in order to be reduced in the following calculation of energy consumption or carbon emissions.

Quantity of electricity and fuel are the most important indicators in the operation stage. The relative data is operated and maintained by the property management company and is also exchanged with the database based on a real-time basis.

In the stage of demolition, the data of transportation distance of auxiliary materials, machinery and equipment are maintained by the demolition company as well as the amount of electricity and fuel used in office and living areas. Furthermore, if the auxiliary materials are used only once on site and cannot be recycled for further use, the quantity is also recorded to calculate the energy consumption and carbon emissions. If the materials can be recycled, the corresponding quantity is also recorded to be reduced in the following calculation.

## 4. Concept of Model Design

### 4.1. Adopting RFID to Track Building Materials in Their Life Cycle

RFID provides a flexible, reliable and efficient way for transmitting product information, which has mainly been considered to be the identity, using radio waves. With the cost of RFID tags being reduced significantly in the last few years, the technology looks set to become the key technology in a new era of product identification and tracking. Building materials are also products [39], therefore they are not exempt from the forthcoming massive RFID adoption. Actually, RFID technologies have already been linked with construction and building management application in the past decade.

As a technology promoted mostly in the manufacturing and logistics area, it is not surprising that RFID has drawn the attention of researchers and business in the field of building material production/manufacturing. Schultmann and Sunke suggested that RFID in the building material production stage could help achieve efficient identification and accurate shipments of products [40]. Later some building materials manufacturers have also adopted RFID on their products for the purpose of fighting counterfeiting, completing projects on time and within budget, and improving on-site safety [41].

However, most of the research that uses RFID for building materials lies at the construction stage. Jaselskies and El-Misalami conducted the pioneering study using RFID for construction process management and concluded that the technology is useful, reliable, robust, and secure and has the potential of facilitating construction processes [42]. Song *et al.* carried out field tests to determine the technical feasibility of using RFID for automatically identifying and tracking individual pipe spools in laydown yards and under shipping portals [43]. The result indicated that the technology could function effectively in the construction field environment involving large metal objects and requiring relatively long read range. A more extensive study was presented later in [44], in which the author tried to find out the optimal frequency range and material to apply RFID to building materials. In the study a comprehensive performance test result of various RFID technologies on typical building materials, such as steel, stone, window, glass, and finishing material, were presented in order to find the best location and tag type. Such result suggests that it is feasible to tag building materials, though the tag type and attachment method needs to be carefully selected based on the type of the material. Some preliminary system implementation methods were then proposed in Ren *et al.*, in which the authors investigated how RFID technology could be integrated with the existing construction material management system seamlessly [45]. Sardroud later conducted research at the construction management level that used RFID with combined GPS and GSM devices to identify and track construction materials [46]. The author concluded that as a powerful portable data collection tool, RFID enables the collection, storage, sharing, and reuse of field data accurately, completely, and almost instantaneously.

Moreover, the operation/maintenance stage and demolition/deconstruction stage have not been ignored by the research community. Schultmann and Sunke recognized the benefits of having RFID-tagged building materials in both their operation/maintenance and demolition/deconstruction stages [40]. Recent research by Park and Kim demonstrated that not only can RFID tags be attached to building materials, and they could also be implanted in them [47]. In the reading performance measurement with RFID tags buried in concrete bricks, although the reading distance was significantly reduced, the reading function was successfully achieved. The positive result of such feasibility study underpins the extension of RFID adoption into the full life cycle of building materials.

### 4.2. Carbon-Emission Data Storage on Tags

As discussed in the previous section, both the desire of using RFID systems and the underpinning technologies for RFID tags to be attached with building material products already exist. In certain life cycle stages, pioneering adoptions have been presented in real industry applications. However, in almost all the existing cases, RFID technologies have been used simply as a replacement of barcodes and are considered merely as an automatic input method, which means the data contained in the RFID tags is just the static identification. In our research, we move a step further on the usage of the RFID tags as a dynamic data carrier. The essential idea is that RFID tags are considered not only a static identity carrier, but also a medium to store and transmit dynamic customized information.

For a long time the dominant RFID system architecture has been the so called “data-on-network”, which was proposed by EPC Global as the main architecture of RFID based network systems. In such architecture the RFID tags carry only an identity and all object related data is saved in the EPC network. However, since 2010 EPC has allowed customized “user data” to be written in class 2, 3 and 4 RFID tags. This enhanced RFID tags functionality and led to the concept of “data-on-tag” that enables the design of our system architecture. Diekmann *et al.* discussed both architectures in general and concluded that they both have their own advantages and that they can be used in an integrated approach [48]. As EPC standards did not define the specifics of the syntax, semantic and serialization of the “user data”, Tribowski *et al.* suggested an adoption of ISO 13584 standards for parts libraries (PLIB), with data attributes, data type and unit to be defined in addition [49]. In a later study, Pais and Symonds demonstrated the writing of such data into real RFID tags in XML and CSV serializations [50]. Those works have demonstrated the technical feasibility of using passive RFID tags as customized data carriers.

In our design, we adopt the data-on-tag concept to record on the RFID tags the carbon emission information relevant to each specific unit of building material. This is achieved by recording every activity carried out to the material and its corresponding carbon emission figure in the RFID tag's “user data” memory. Such information could then be deemed as an accurate “carbon footprint” record of the material, and should be updated at each check point, where the tag is to be accessed by a RFID reader device, during the material's life cycle. In addition to just reading out the identity stored in the tag, the reader device at each check points would read out the recorded “carbon footprint” and also update it with any available new entries.

### 4.3. Data Collection/Update with RFID Readers and Data Aggregation with Networked RFID Systems

RFID technology on its own just performs the information transmission between tags and reader at check points. The real potential of such information can be extracted only when it is supported or been used to support a larger system that maintains a central view of the scenario/application. This requires the RFID reader devices to be integrated with an information management system and to be capable of retrieving data from or injecting data into the system's network backbone. Such integration usually leads to the so-called “Networked RFID System”. In fact, network device and platforms have long been identified as a significant part of RFID system spending, together with tags and readers [51].

Many network level implementations were proposed in the past few years that have seen RFID systems integrated with most of the popular network protocols, both wired and wireless. Each of these implementations has its own features that may suit applications in one or several different stages of building materials' life cycle. In the production stage the material manufacturers are normally large plants with existing networks, such as local area networks or fieldbus, for automation and control systems. Therefore the best way to deploy a RFID system would usually be integration with the legacy network systems. On the other hand, the construction stage presents a much more challenging scenario due to its dynamic and uncontrolled operational environment, in which most of the traditional networking technologies struggle to work properly. Our proposed/recommended integration method for this stage is the RFID Sensor Networks, which adopts a flexible wireless sensor network backbone to deploy various types of RFID systems in construction sites. Such a method has been demonstrated in our previous research [23]. Moreover, most modern intelligent buildings are equipped with building/home automation systems. Therefore for operation stage and demolition stage, the RFID systems could be easily integrated into the network of those intelligent systems. Such integration could be done in traditional TCP/IP network [52] as well as RFID sensor networks [53,54].

### 4.4. Data Sharing with a Heterogeneous Network Architecture

**Figure 3.**
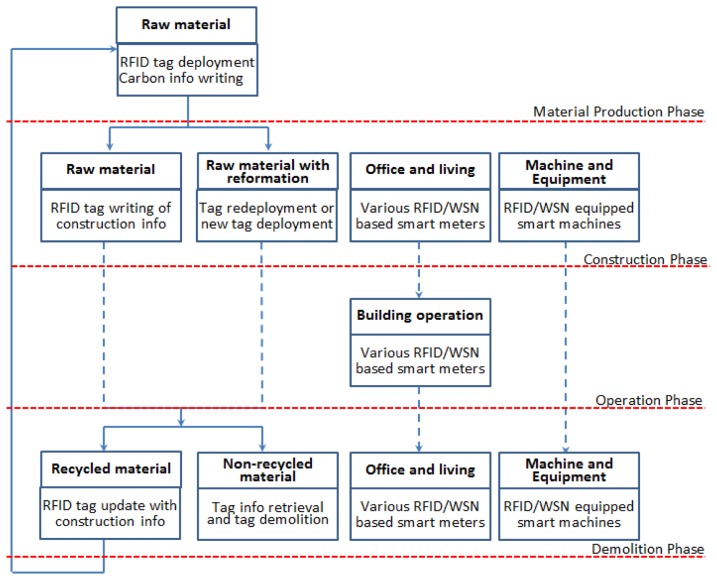
The connection between the design concept and Figure 2.

Based on the discussion in the previous section, we believe that it is neither sensible nor necessary to enforce a unified network protocol or structure for the local integration of network RFID systems. We recognized that each particular application/party, which the building materials passed through at some point of their life cycle, could look at their specific requirements and have their own integration method of choice. We believe that as long as the RFID systems could be integrated to perform data reading/writing operation and that the application information network could provide a gateway to share and accept the information related to their data, the whole architecture we proposed will not be affected by the specific network integration method chosen by individual party participated. In this case, the virtual network that connects the systems of all stakeholders will be in the form of a heterogeneous network that connects many different types of networks and devices. Figure 3 illustrates the connection between design concept and Figure 2.

## 5. System Architecture

**Figure 4.**
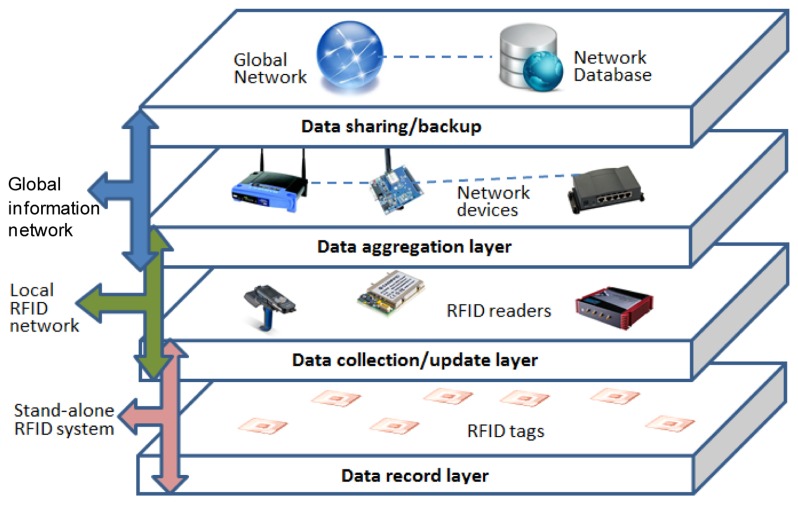
RFID-based carbon emission recording/tracking system architecture.

Based on the basic design concepts we present architecture for a RFID-based carbon emission recording/tracking system in Figure 4. Such a system architecture contains four functional layers, which are: data record layer, data collection/update layer, data aggregation layer and data sharing/backup layer. Each of these layers is formed by RFID or network devices and sub-systems that operate at a specific level.

The data record layer contains the RFID tags carrying object identity and related carbon emission information. The main function of this layer is to maintain data records at the “tag/object level”. Therefore it has upwards responsibility to make the tag data accessible to the upper layer devices and to accept and record new entries from them.

Data collection/update layer mainly contains the RFID reader devices. Its main functionalities include communicating with the RFID tags and pushing/receiving information to its devices' hosting systems, which are located in the next layer. With devices operating at “terminal level”, this layer is the boundary of the networked and non-networked parts of the system and the devices in the layer can be deemed as the “data contact/touch points”/“hands” of the system that retrieve or manipulate the non-networked data on RFID tags. This also means the layer has upwards responsibility to make the devices in the layer network-ready, which means that they should be able to be hosted in the network systems in the next layer either by having their own embedded network modules or by connecting to computing devices.

The data aggregation layer involves the network devices that are used to maintain the local RFID network systems. Examples of devices in the layer include local network servers, routers and computing devices that the RFID readers are connected to if they are not embedded with network modules themselves. The functionality of the layer is mainly to provide application-friendly local RFID networks that host the reader devices and aggregate the data collected by them at the “application level”. As discussed in Section 4.1, the structure and protocol of the local system in this layer do not affect our full architecture and therefore the systems in the layer could be heterogeneous. The local systems should be designed to suit the specific application requirements of each stakeholder. The layer also has upwards responsibility to make its data available in standard format to the upper layer through interfaces on gateway device connected to the global networks.

This leads to the top level data sharing/backup layer that concerns the data's availability at a “global level”. As discussed in Section 3.2 this could involve a centralized or cloud database maintained by some authority department, construction association or authorized non-government organizations. It is clear that by having the top level layer we are adopting a combination of “data-on-network” and “data-on-tag” architectures. The advantage of such a design is that it ensures the safety/integrity of information by having the extra data redundancy to tackle the inevitable issue of having broken tags during the life cycle of the building materials, while at the same time still allowing stand-alone reader devices and processes. The later means the local devices do not need to be fully managed online as long as certain level of synchronization is supported to maintain the data integrity at all levels.

One of the crucial points for the successful implementation of such architecture is a unified, effective and standardized data format to support the information passing between the different layers. Using RFID tags as data storage requires a unified data representation and semantic. As we expect most of the building materials to be passed through multiple parties throughout their life cycle, the application can only work if all parties involved adopt unified data syntax. Instead of inventing an entire set of new specifications, there are existing standards that we can build on. Tribowski *et al.* proposed the use of ISO 13584 for parts libraries (PLIB) [49]. In addition, there are also standards in the ISO 14000 family concerning carbon footprints, such as ISO 14064 and ISO 14067, which can help build the data attributes, data type and unit required. The data serialization could take any standard format that is currently used for network data sharing, such as XML, CVS and JSON, so that we could have a unified serialization method from tag to the global networks. However, considering the limited user memory space in passive RFID tags, the serialization format with a lighter weight might be preferable.

Such an architecture provides a possible design for recording and maintaining carbon emission data on RFID tags. As RFID tag identifies a specific item rather than just the type, the carbon emission data can be recorded for activities carried out on each specific unit of material. As RFID tags can follow the material through its full life cycle, we believe that this will significantly improve the accuracy and reliability of carbon emission calculation for building life cycle.

## 6. Illustration of an Implementation and Demonstration System

We developed a proof-of-concept system to illustrate our proposed architecture and demonstrate the feasibility of the design. The system consists of a CAEN A528 UHF passive RFID reader module, five Jennic JN5148 ZigBee wireless modules, a local gateway, a corporate server and a remote client computer. A few UHF passive RFID tags were also used and attached to several different types of building materials. Those tags are considered to be in the data record layer and initially carry the identity of the materials they are attached to. For the data collection layer the RFID reader module was integrated with one of the ZigBee wireless modules via a serial UART connection. From the network system's point of view, this merges the two modules into a single ZigBee network ready RFID reader. Another ZigBee module acts as the coordinator of the network and also as a sink node of the sensor network by connecting to the gateway computer via RS232 connection. The remaining ZigBee modules form the backbone of the local ZigBee wireless network that accommodates all the devices. For the data aggregation layer we also implemented a local gateway on a raspberry-pi computer, which is a low cost and energy efficient solution suitable for long-term deployment during the building operation phase as well as the other relatively short-term phases. The gateway computer retrieves information from the serial connection with the ZigBee sink node and serialize it into standard format that's ready to be injected into the data sharing layer, for which we implemented services on the gateway computer, the corporate server and the remote client computer to illustrate how data can be shared both within the corporation and to third parties. The core service in this system lies on the corporate server, which is developed as a Sensor Web Enablement (SWE) server complied with the Open Geospatial Consortium's Sensor Web Enablement (OGC-SWE) specifications to provide the initiative and illustration of using standard format and interfaces. Its core is based on part of 52North's sensor web open source releases and we managed to host a data storage with internal data model and a standard RESTful Web interface for both data input and output on the server. On the gateway computer we implemented agent software to enable it to make standard queries to the corporate SWE server to perform registration of new sensor, RFID reader or RFID tags as well as insertion of the data retrieved from them in a standardized XML serialization. We then put a simple Web application to request data interested on the remote client computer, which illustrates either a third party or a regulation body's server that is interested in the published information.

**Figure 5.**
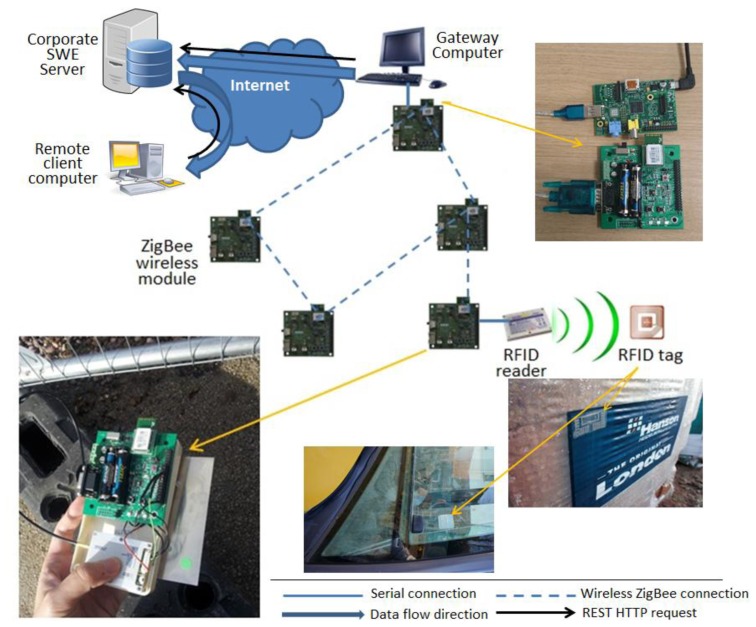
Demonstration system structure and implementation.

Figure 5 shows the system deployment of this experiment. On initialization phase the corporate server is first started with the SWE server services online. The gateway computer(s) then goes onlive and registers themselves with the corporate server. After that the ZigBee coordinator establishes the sensor network, followed by the joining of the other four modules, all of whom act as ZigBee routers. The ZigBee RFID reader device then starts working and reports back to the coordinator, who sends updates to the gateway computer regularly. In this particular demonstration system the new RFID reader is identified in the updates therefore the gateway computer registers the reader as a new sensing device to the corporate server, where the it can then be assigned a roll such as an access point, a driver's mobile reader or even a smart meter. Any tag reading and its accompanying information (e.g., transport distance of and activities performed on the piece of material represented by the tag read by a driver's or access point's reader) from it afterwards is then updated to the gateway device and injected to the corporate SWE server by making REST HTTP queries with the data in XML serialization complied to the SWE specifications. The information injected and stored on the SWE server then undergoes predefined process, in our case a conversion of some received information to carbon emission figure. The remote client computer can then send standard SWE-complied REST HTTP queries to the corporate server to request any information that it is interested in. To illustrate the building life cycle we are able to perform identity retrieving on wood and concrete materials and construction machines, activity and carbon emission data writing and third party querying, which are the common tasks that can happen in all phases in the cycle. We believe such demonstration system shows the technical feasibility of our proposed architecture and a possible way of implementation, for which other methods of implementing data modelling and serialization exists, such as W3C-SSN and JSON *etc*., but the system architecture remains as what we have proposed regardless of which implementation methods is adopted.

To illustrate the underlying route of improving calculation accuracy, the formula used in [5] is listed and analyzed as follows:

(1) $$E^{\text{manu}}=\sum_{i=1}^{9}Q_{i}^{\text{cons}}e_{i}^{\text{cons}}(i=1,2,\cdots ,9)$$

where *E*^manu^: energy consumption in building materials production stage; $Q_{i}^{\text{cons}}(i=1,2,3,\cdots 9)$ : quantity use of building material (diesel fuel, electricity, concrete, cement, brick, steel, timber, glass, plastic) during construction stage; $e_{i}^{\text{cons}}(i=1,2,\cdots ,9)$ : energy consumption of manufacturing per weight of building materials.

(2) $$E^{\text{cons}}=S_{m}^{\text{cons}}d(1+1/2)+T^{\text{cons}}(m=1,2,3,\cdots ,7)$$

where *E*^cons^: energy consumption in building construction stage; $S_{m}^{\text{cons}}(m=1,2,3,\cdots ,7)$ : transport distance of building material (concrete, cement, brick, steel, timber, glass, plastic) from the material manufacturing site to construction site; *d*: energy consumption per building material and per transport distance; *T^cons^*: energy consumption during construction-installation on-site processes.

(3) $$E^{\text{oper}}=(Le^{\text{oper}}+Lh^{\text{oper}})e^{\text{oper}}$$

where *E*^oper^: energy consumption in building operation stage; *Le*^oper^: electricity use for cooling, ventilating, lighting, and water supply during operation stage; *Lh*^oper^: electricity use for heating during operation stage; *e^oper^*: conversion factor of electricity to energy.

(4) $$E^{\text{demo}}=\sum_{j=1}^{7}\left[Q_{j}(1-r_{j})]S^{\text{demo}}d+T^{\text{demo}}(j=1,2,3,\cdots ,7\right)$$

where *E*^demo^: energy consumption in building demolition stage; *Q_j_*(*j* = 1,2,3,⋯,7): quantity use of building material (concrete, cement, brick, steel, timber, glass, plastic); *r_j_*: recycle rate of building material; *S^demo^*: transport distance of building material from the construction site to landfill site; *T^demo^*: embodied energy of diesel fuel consumed for deconstruction.

As our design model and the proposed architecture can significantly improve the recording accuracy of $Q_{i}^{\text{cons}}$, $S_{m}^{\text{cons}}$, *Le^oper^, Lh^oper^, Q_j_* and *S^demo^*, it can consequently improve the accuracy of energy consumption, which is also the same in references such as [1] and [2] and so on. In a word, our design model and the proposed architecture can fill the extant gaps of accurately recording, real-time transferring and sharing of key indicators in the calculating process of energy consumption and carbon emissions of sustainable buildings based on a RFID-based system.

## 7. Discussion and Conclusions

Building sustainability is one of the crucial parts of achieving urban sustainability. Applied to buildings, life-cycle assessment encompasses the analysis and assessment of the environmental effects of building materials, components and assemblies throughout the entire life of the building construction, use and demolition. Estimate of carbon emissions are essential and crucial for an accurate and reasonable life-cycle assessment.

Addressing the need for more research into integrated analysis of real-time and automatic recording of key indicators for a more accurate calculation and comparison, this paper aims to design a real-time recording model of these crucial indicators concerning the calculation and estimation of energy use and carbon emissions of buildings based on a RFID-based system. The architecture of the RFID-based carbon emission recording/tracking system is presented, which contains four functional layers including data record layer, data collection/update layer, data aggregation layer and data sharing/backup layer. Each of these layers is formed by RFID or network devices and sub-systems that operate at a specific level. Finally, a proof-of-concept system is developed to illustrate the implementation of the proposed architecture and demonstrate the feasibility of the design. This system consists of a RFID reader module, ZigBee wireless modules and several computers and servers to show a possible way of developing and deploying such an architecture. We've chosen to use services and data modelling and serialization that comply with the OGC-SWE, which is the leading specification aiming to integrate sensing and geographical information with the Web and Internet of Things. We recognize that there are a few other ways of implementing data models and serialization but adopting a different one does not affect the system architecture we proposed. This study is set to provide the technical solution for a real-time recording system of key calculation and estimation indicators of building energy use and carbon emissions and thus is of great significance and importance in improving urban sustainability.

## Figures and Tables

**Table 1.** Estimation of key indications of calculation in recent research.

**Source**	**Key Indicators of Calculation (Energy Consumption or Carbon Emission)**	**Estimated or Not**	**Real-Time Based or Not**
[5]	Quantity use of building materials, fuel and electricity. Transportation distance of building materials.	Estimated	Not
[1]	Quantity use of building materials, fuel and electricity.	Estimated	Not
[2]	Quantity use of building materials, fuel and electricity.	Estimated	Not
